# An Improved MLVF Method and Its Comparison with Traditional MLVF, *spa* Typing, MLST/SCC*mec* and PFGE for the Typing of Methicillin-Resistant *Staphylococcus aureus*

**DOI:** 10.3390/ijms15010725

**Published:** 2014-01-08

**Authors:** Xue-Fei Du, Meng Xiao, Hong-Yan Liang, Zhe Sun, Yue-Hong Jiang, Guo-Yu Chen, Xiao-Yu Meng, Gui-Ling Zou, Li Zhang, Ya-Li Liu, Hui Zhang, Hong-Li Sun, Xiao-Feng Jiang, Ying-Chun Xu

**Affiliations:** 1 Department of Clinical Laboratory, the Fourth Affiliated Hospital of Harbin Medical University, No. 37, Yiyuan Street, Nangang, Harbin 150001, Heilongjiang, China; E-Mails: duxuefei82@163.com (X.-F.D.); lhyhydsy@126.com (H.-Y.L.); sunsz00000@163.com (Z.S.); jiangyuehonghydsy@126.com (Y.-H.J.); cgyhydsy@126.com (G.-Y.C.); mxyhydsy@126.com (X.-Y.M.); zglhydsy@126.com (G.-L.Z.); 2 Department of Clinical Laboratory, Peking Union Medical College Hospital, Chinese Academy of Medical Sciences, No. 1, Shuaifuyuan, Dongcheng, Beijing 100730, China; E-Mails: xmpumch@126.com (M.X.); zlpumch@126.com (L.Z.); lylpumch@126.com (Y.-L.L.); zhpumch@126.com (H.Z.); shlpumch@126.com (H.-L.S.)

**Keywords:** methicillin-resistant *Staphylococcus aureus* (MRSA), genotyping, multilocus variable-number tandem repeat fingerprinting (MLVF), capillary gel electrophoresis, pulse field gel electrophoresis (PFGE), Multilocus sequence typing (MLST), staphylococcal cassette chromosome *mec* (SCC*mec*)

## Abstract

Methicillin-resistant *Staphylococcus aureus* (MRSA) has become an important nosocomial pathogen, causing considerable morbidity and mortality. During the last 20 years, a variety of genotyping methods have been introduced for screening the prevalence of MRSA. In this study, we developed and evaluated an improved approach capillary gel electrophoresis based multilocus variable-number tandem-repeat fingerprinting (CGE/MLVF) for rapid MRSA typing. A total of 42 well-characterized strains and 116 non-repetitive clinical MRSA isolates collected from six hospitals in northeast China between 2009 and 2010 were tested. The results obtained by CGE/MLVF against clinical isolates were compared with traditional MLVF, *spa* typing, Multilocus sequence typing/staphylococcal cassette chromosome *mec* (MLST/SCC*mec*) and pulse field gel electrophoresis (PFGE). The discriminatory power estimated by Simpson’s index of diversity was 0.855 (28 types), 0.855 (28 patterns), 0.623 (11 types), 0.517 (8 types) and 0.854 (28 patterns) for CGE/MLVF, traditional MLVF, *spa* typing, MLST/SCC*mec* and PFGE, respectively. All methods tested showed a satisfied concordance in clonal complex level calculated by adjusted Rand’s coefficient. CGE/MLVF showed better reproducibility and accuracy than traditional MLVF and PFGE methods. In addition, the CGE/MLVF has potential to produce portable results. In conclusion, CGE/MLVF is a rapid and easy to use MRSA typing method with lower cost, good reproducibility and high discriminatory power for monitoring the outbreak and clonal spread of MRSA isolates.

## 1. Introduction

Methicillin-Resistant *Staphylococcus aureus* (MRSA) is a dangerous human pathogen, causing considerable morbidity and mortality worldwide. In China, the prevalence of MRSA is high (40%~80%) [1,2], and MRSA controlling has been made a high priority among health care professionals [3]. Important components of control strategies are detecting the resistant strains and tracking of the transmission of strains in hospitals and the community by effective molecular typing. Currently, several different molecular typing methods are available [4]. However, each method has its own strengths and weaknesses, and no single method yet combines high discriminatory power, full inter-laboratory portability, ease of performance and low cost.

Up to now, the gold standard for short-term epidemiological surveillance of *S. aureus* has been pulsed-field gel electrophoresis (PFGE) [5,6], although some new typing methods were reported as having higher or similar discriminatory power compared to PFGE [7–10]. However, PFGE is labour intensive and costly. In addition, the interlaboratory comparison of data is challenging. Multilocus sequence typing (MLST) is an ideal method for long-term epidemiological studies, but its routine application is very unfeasible in clinical laboratories. Staphylococcal cassette chromosome *mec* (SCC*mec*) is a useful method to differentiate community-associated (CA) MRSA from hospital-acquired (HA) MRSA. However, as a typing method, SCC*mec* should not be used alone. Recently, *spa* sequence typing has become the most popular typing method for *S. aureus*. Genetic diversity in the *spa* locus arises from both a Variable Number of Tandem Repeats (VNTRs) and point mutations in the gene encoding the cell surface protein A. Although *spa* typing showed less discriminatory power than PFGE [11], its low cost, high reproducibility, appropriate stability, high throughput, and full data portability made this method the primary tool for characterization of MRSA isolates at the local and international level [3,12]. However, *spa* typing also has certain limitations. The method can misclassify particular types due to recombination/homoplasy and there are no clear rules as to which cost of the BURP algorithm should be used during a study.

In 2003, a new method for typing *S. aureus* strains, multilocus variable number tandem repeat fingerprinting (MLVF), formerly called the multilocus variable-number tandem-repeat analysis (MLVA), was applied [13]. In the present study this method is referred to as ‘traditional MLVF’ so as to differentiate it from our improved MLVF method. Traditional MLVF analyzes polymorphisms of VNTRs regions located in seven genes (*sspA*, *spa*, *sdrC*, *sdrD*, *sdrE*, *clfA and clfB*). The discriminatory power of traditional MLVF was found to be comparable with that of PFGE and higher than that of MLST, other MLVA methods, *spa* typing and other PCR-based methods [11,14–16]. In addition, traditional MLVF was reported which has the discriminatory power needed to rapidly distinguish very similar community-acquired and nosocomial MRSA isolates [17]. However, traditional MLVF is based on conventional agarose gel electrophoresis, which limits its reproducibility and accuracy. In addition, analysis based on comparing multiple banding patterns on agarose gels are not easily comparable between laboratories, which precludes the constitution of international databases. Another VNTR-based typing method MLVA, using Capillary Gel Electrophoresis (CGE) instead of agarose gel electrophoresis, produces more accurate data than traditional MLVF, and allows the production of the genotype in the form of a code that can be stored in a database and easily shared and compared with other laboratories. However, there is still no consensus on which one is the best scheme or which set of markers should be used.

In 2012 Sabat, *et al.* [18], the developer of traditional MLVF, established a new method for typing MRSA with newly designed set of primers for the same VNTR regions of traditional MLVF and combined it with micro-fluidic chip. This new set of primers was considered to obtain more stable data. However, our validation test showed the newly designed set of primers was not as stable as the original set of primers. Also, the micro-fluidic chip is still an expensive technique and is not commonly used in China today. Therefore, the present study was designed to improve the resolution of traditional MLVF by combining its PCRs scheme with Capillary Gel Electrophoresis, and here we named this improved method as CGE/MLVF. Moreover, criteria for clustering of CGE/MLVF patterns were proposed based on comparison with the data produced by *spa* typing, MLST and PFGE.

## 2. Results

In the present study, traditional MLVF was improved by combination multiplex PCR with capillary gel electrophoresis instead of traditional agar gel electrophoresis in order to obtain better discriminatory power and reproducibility.

### 2.1. Multiplex PCRs Design and Pattern Profile Definition

In this study, all the 42 well characterized strains and 116 clinical isolates were first typed by traditional MLVF. Comparing all the amplicon bands in agarose gels to DNA markers, we determined the size range of every VNTR amplicon of all the isolates. Considering the size range of each VNTR amplicon and the abundance of each loci amplicon, we labeled five upstream primers with different fluorescence and designed two PCR panels (see Table 1). The concentrations of each primer within the same panel were optimized to obtain optical fluorescence intensity. For each isolate, the sizes of each VNTR loci in both two panels comprised the pattern profile (see Table 2).

### 2.2. CGE/MLVF Typeability

Two multiplex PCRs with different fluorescently labeled primer sets made it possible to distinguish bands of different VNTR loci and produce a profile for every isolate. For well-characterized strains the typeability of CGE/MLVF was 100%. For clinical isolates the typeability of CGE/MLVF was 99%, because VNTR locus *clfA* did not yield a band against clinical isolate 1466. Further analysis of this isolate at this locus by single PCR and agarose gel electrophoresis revealed a band of ~1400 bp, which could not be detected by using capillary electrophoresis, since it was larger than the upper detection limit of the LIZ marker.

In CGE/MLVF PCR panel 1, loci *clfA* and *clfB* yielded only one band respectively in all isolates. The *sdr* locus yielded one to three bands, because its gene comprises two or three closely linked and tandemly arrayed open reading frames containing *sdrC*, *sdrD* and *sdrE*. For 116 clinical isolates, the *Sdr* locus yielded three bands for each isolate except for isolate 965, for which it yielded two bands. For 42 well-characterized strains, the *sdr* locus yielded three bands against 34 strains, two bands against five strains and only one band against three strains. Of these, reference strains Mu3, Mu50, USA300, COL and MW2 were all detected, yielding three bands from the *sdr* locus, which is in agreement with the data in the NCBI database.

**Figure 1.**
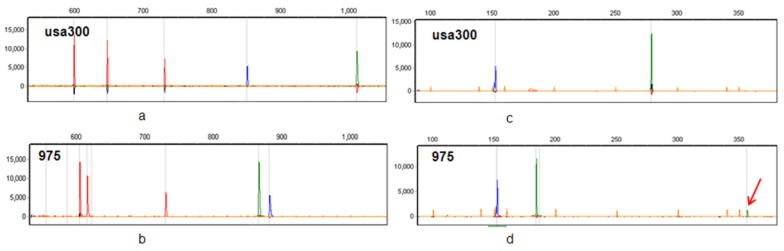
The CGE/MLVF electrophoresis figures of USA300 and clinical isolate 975. For CGE/MLVF panel 1 figure of USA300 (**a**) and clinical isolate 975 (**b**); red peak represents *sdrCDE*, green peak represents *clfA* and blue peak represents *clfB*. For CGE/MLVF panel 2 figure of USA300 (**c**) and clinical isolate 975 (**d**); blue peak represents *sspA*, green peak represents *spa*. Red arrow in figure 1d shows the nonspecific band of *spa*.

In CGE/MLVF PCR panel 2, locus *sspA* yielded only one band for all isolates. However, for most isolates the *spa* locus yielded two bands, a smaller one and a larger one. The larger band was about 171 bp longer than the smaller, and was detected with 5%~20% of the fluorescence intensity of the smaller band (see Figure 1d). Further comparison of the *spa* amplicons of reference strains COL, MW2, USA300 and Mu3 with data in the NCBI database confirmed that the large weak band was nonspecific; in addition, the fluorescence intensity of the *spa* nonspecific band was less than 10% of the specific band as a whole. Therefore, we ignored this nonspecific band in the present study.

In brief, the number of specific bands detected in both two panels for each isolate varied between five and seven. The CGE/MLVF electrophoresis figure of two panels of USA300 and clinical isolate 975 (belong to CGE/MLVF cluster a) were shown in Figure 1.

### 2.3. The Resolution of CGE/MLVF against Well-Characterized MRSA Strains

Forty-two well-characterized strains of diverse STs were typed by CGE/MLVF and traditional MLVF. The STs, SCC*mec* types and *spa* types of all the well-characterized strains were previously established. CGE/MLVF showed a better resolution than other typing methods. 15 STs, seven SCC*mec* types and 20 *spa* types were found among the 42 strains, whereas CGE/MLVF yielded 36 distinct types. Different STs shared distinct CGE/MLVF types except for two strains, RPH81 (ST239-III-t037) and AH1 (ST128-III-t037), which shared the same CGE/MLVF type. In addition, CGE/MLVF distinguished between isolates that have the identical ST-SCC*mec*-*spa* types (see Table 2). For example, strain PC8, FH, SJOG30, RPH85, RHH58 and RHH10 were all ST1-IV-t127, but shared four different CGE/MLVF types.

#### 2.3.1. CGE/MLVF Compared to Traditional MLVF

Traditional MLVF yielded the same number types (36 patterns) as CGE/MLVF against the 42 well-characterized strains. However, for some isolates the numbers of amplicons detected by the two typing methods were different. For example, traditional MLVF showed one band less than CGE/MLVF for typing well-characterized strain PC8, RPH85 and SJOG30, because the two amplicons of *sdrCDE* (bands 677 and 683 bp) could not be distinguished by agarose gel electrophoresis. Traditional MLVF could not distinguish bands of *clfA* and *clfB* (993 and 991 bp) against strain RPH81, AH13 and AH1. Altogether, traditional MLVF showed one band less than CGE/MLVF against 10 well-characterized strains and two bands less than CGE/MLVF against two well-characterized strains (Table 2).

### 2.4. Evaluation on Clinical MRSA Isolates

#### 2.4.1. Discriminatory Power

The discrimination indices (DIs) of compared methods were shown in Table 3. The discriminatory power of CGE/MLVF was equal to traditional MLVF. Both two methods were the most discriminatory methods in the present study. In the case of PFGE, the difference was not statistically significant because of overlapping 95% confidence intervals. The discriminatory power of CGE/MLVF was significantly higher than those of *spa* typing and MLST/SCC*mec* (non-overlapping 95% confidence intervals). The isolates with identical CGE/MLVF types were also indistinguishable by *spa* typing and MLST/SCC*mec* typing.

#### 2.4.2. CGE/MLVF

**Figure 2.**
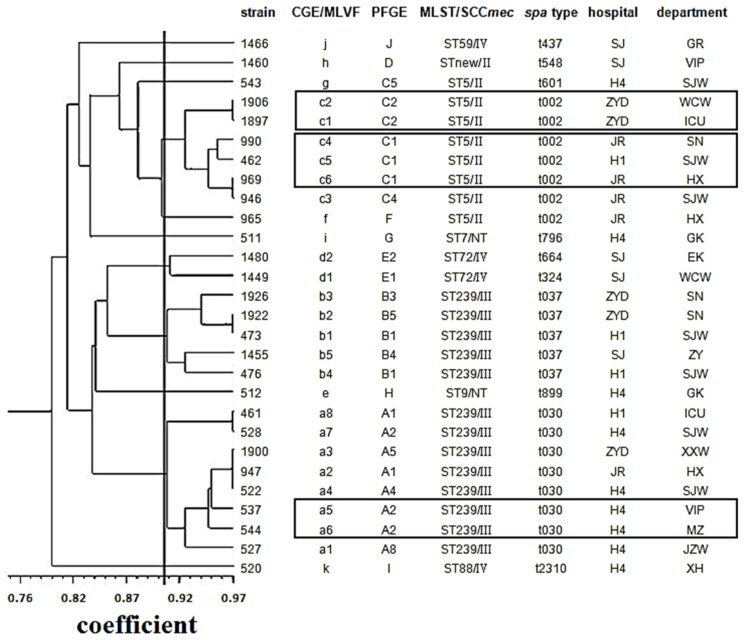
The UPGMA dendrogram based on CGE/MLVF binary matrix showing the relationship between 28 representative clinical MRSA isolates. Boxes in the figure indicate that CGE/MLVF can distinguish isolates with identical PFGE patterns.

Analyzed by GeneMarker software and visual verification, CGE/MLVF produced 28 different patterns among 116 clinical MRSA isolates. Nine patterns were represented by two or more isolates (97 isolates in total). The remaining nineteen patterns contained a single isolate. Figure 2 showed the CGE/MLVF UPGMA dendrogram of 28 representative clinical MRSA isolates and their clustering results by other comparing typing methods.

#### 2.4.3. Traditional MLVF

Analyzed by bionumerics software and visual verification, traditional MLVF provided the same typing results with CGE/MLVF and also separated the 116 clinical MRSA isolates into 28 different band patterns. The clustering results were similar to CGE/MLVF. Comparing to PFGE, the cut-off level of 65% similarity was set up for traditional MLVF to get the best concordance with PFGE.

#### 2.4.4. *Spa* Typing

**Figure 3.**
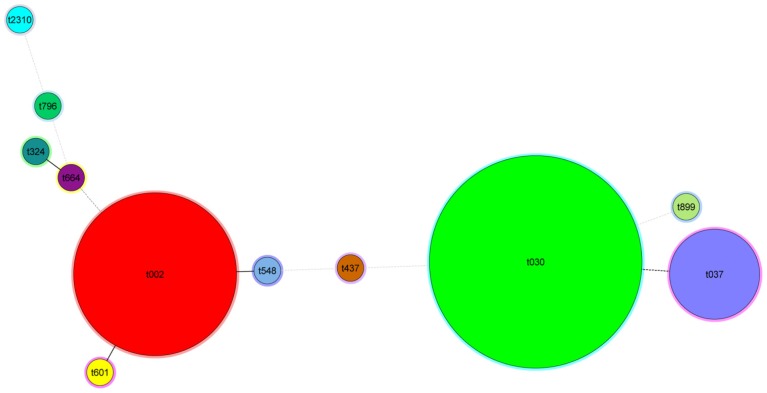
Minimum spanning tree of 116 clinical MRSA isolates typed by *spa* typing. The Spa types are displayed as circles. The size of each circle indicates the number of isolates with this particular type. Thick solid lines connect types that differ in a single VNTR locus and a dotted line connects types that differ in more than 1VNTR loci.

Among all examined isolates, our analysis yielded 11 *spa* types, varying in length between five (t899) and 11 (t2310 and t601) repeats (Table S1). 3 types were represented by 2 or more isolates (108 isolates in total) while eight types contained a single isolate. *Spa* types were clustered using the *spa*-plugin in the Bionumerics software and the results were displayed in a minimum spanning tree (Figure 3).

#### 2.4.5. MLST/SCC*mec*

The 116 clinical MRSA isolates produced eight MLST/SCC*mec* types. Adding SCC*mec* typing did not yield new types compared to using MLST alone. The 116 clinical MRSA include ST239-III (72 isolates), ST5-II (37 isolates), ST72-IV (2 isolates), and one isolate each for ST7-NT, ST9-NT, ST88-IV, ST59-IV and STnew-II type.

#### 2.4.6. PFGE

The 116 clinical MRSA isolates produced 28 PFGE patterns. Analyzed by Bionumerics software and visual inspection using a cutoff value of 84% (equals to six bands difference), 110 of the isolates were classified into 4 clusters, while six isolates had separate positions in the dendrogram.

### 2.5. Reproducibility of All the Typing Methods

In order to test the reproducibility of all the typing methods, 13 isolates were typed in two independent experiments with different DNA preparations, PCR amplifications and running the samples on different agar gels. In these experiments CGE/MLVF showed excellent reproducibility (100%). MLST, *spa* typing and SCC*mec* also showed excellent reproducibility (100%). However, the reproducibility of both traditional MLVF and PFGE were challenging when images were analyzed by Bionumerics software alone without visual inspection (both less than 70%). With visual inspection the reproducibility of traditional MLVF and PFGE were 100% and 92% respectively. DNA band location using traditional MLVF was usually influenced by running on different gels even under strict quality control. A certain isolate’s typing in two independent experiments with different PCR amplifications and running on different agar gels sometimes caused different clustering using Bionumerics software. For PFGE, the band location and shape were always different by running in different agar gels and also caused false clustering.

### 2.6. Criteria for Defining CGE/MLVF Clusters

In order to determine the rules for clustering the patterns into clonal groups, different similarity coefficients were tested. We searched for a level of similarity between CGE/MLVF clusters that would be in best concordance (AR) with the determined PFGE clonal complexes (with cut-off value of 84%, CI84%) and PFGE patterns. On visual inspection of the CGE/MLVF dendrogram, we tested the similar coefficient 0.905, 0.925 and 0.945. The concordance (AR) for CGE/MLVF clusters and PFGE clonal complexes was 0.989, 0.684 and 0.577, respectively. Therefore, the CGE/MLVF clusters were most consistent with PFGE clonal complexes when the similar coefficient between CGE/MLVF banding patterns was set at 0.905. With the similar coefficient of 0.905, 109 of the clinical isolates were classified into four clusters designated Ca to Cd, while seven isolates had separate positions in the dendrogram.

### 2.7. Concordance between CGE/MLVF, *spa* Typing, MLST/SCC*mec* and PFGE

The concordance values between the typing methods compared were listed in Table 4. The highest and satisfied concordance was found when the CGE/MLVF clusters with similar coefficient of 0.905 and *spa* types and PFGE clusters were compared (AR, 0.988 and 0.989, respectively). The concordance between CGE/MLVF and other comparing methods on cluster level was good (see Figure 2). On the pattern/type level, the concordance between CGE/MLVF types and *spa* types, MLST/SCC*mec* and PFGE patterns was much lower (AR, 0.438, 0.308 and 0.384, respectively). This was not surprising, because the discriminatory ability of CGE/MLVF was much higher than those of *spa* typing and MLST/SCC*mec*. Holmes *et al.* also showed a lower concordance between traditional MLVF and PFGE patterns [14].

CGE/MLVF both on the level of types and clusters defined by similar coefficient of 0.905 showed complete probability (*W*, 1) to predict the corresponding PFGE clusters, *spa* types and MLST/SCC*mec* types (Table 4). Also the PFGE pattern and cluster had a high probability to predict the *spa* types (*W*, 1 and 0.986) as well as predict CGE/MLVF clusters defined by similar coefficient of 0.905 (*W*, 1 and 0.986). *Spa* type and MLST/SCC*mec* type could be predicted well by PFGE pattern, PFGE cluster, CGE/MLVF pattern and CGE/MLVF cluster. The lowest Wallace’s coefficients were found for PFGE cluster, MLST/SCC*mec* and *spa* type to predict CGE/MLVF patterns (*W*, 0.385, 0.301 and 0.385, respectively).

### 2.8. CGE/MLVF Can Distinguish Isolates Belonging to the Identical Epidemic Clone in China

ST239-III-t030, ST239-III-t037 and ST5-II-t002 were predominant epidemic MRSA clones. CGE/MLVF differentiated 61 clinical isolates of ST239-III-t030 type into 8 CGE/MLVF types, 11 ST239-III-t037 type isolates into 5 CGE/MLVF types and 36 ST5-II-t002 type isolates into 6 CGE/MLVF types.

### 2.9. CGE/MLVF Can Distinguish Isolates with Identical PFGE Patterns

Figure 2 showed that isolates 1906 and 1897 had identical PFGE patterns, but shared different CGE/MLVF types. The two isolates were collected from the same hospital but different departments. In addition, isolates 990, 462 and 969, collected from different hospitals or departments, were all clustered to PFGE C1, but shared CGE/MLVF type c4, c5 and c6, respectively. These data demonstrated that CGE/MLVF could differentiate isolates with identical PFGE patterns.

## 3. Discussion

It is of paramount importance to develop an efficient strategy to prevent the dissemination of MRSA clones and to provide optimal treatment for patients. However, in many hospitals, resources are not available for PFGE or MLST chosen in epidemiological studies. In 2003 the VNTR typing method for typing *S. aureus*, MLVF, was firstly applied. Afterwards, another VNTR-based typing method MLVA was developed [19,20]. However, there is still no consensus on which one is the best method or which set of markers should be used. Holmes *et al.* [14] compared the traditional MLVF and MLVA [19] and considered traditional MLVF was higher discriminatory than MLVA. The discriminatory power of traditional MLVF was also found to be higher than that of MLST, *spa* typing and other PCR-based methods [11,15,16]. However its repeatability and comparability between laboratories were still challenged. Therefore, in the present study we used the set of markers of traditional MLVF and improved the resolution by combining the method with capillary gel electrophoresis (CGE), and named this improved method CGE/MLVF.

We demonstrated that CGE/MLVF was more suitable than traditional MLVF for typing MRSA isolates. CGE/MLVF provided more accurate data than traditional MLVF, since the resolution of CGE was superior to conventional agarose gel electrophoresis. Conventional agarose gel electrophoresis could not distinguish well between bands that differed by less than 15~20 bps. Therefore it could cause false typing results when two isolates yielded bands of similar size. CGE/MLVF, however, showed good performance because of the high discriminatory power of CGE which can distinguish between bands that differ by only 1 bp. In addition, traditional MLVF could not visualize bands of a low abundance PCR product as clearly as CGE/MLVF does, which may also cause false clustering. Moreover, the reproducibility of CGE/MLVF was perfect. The size of the PCR amplicon in two different tests did not differ by more than 1 bp. However, traditional MLVF based on conventional gel electrophoresis, was always affected by factors, such as gel concentration and other uncontrollable factors in different tests, and showed poor reproducability.

Data analysis for CGE/MLVF was straightforward, since the fragment sizing is automated and genotype profiles are obtained. However, traditional MLVF data analysis is subjective and ambiguous. Binumerics greatly facilitated the analysis of traditional MLVF banding patterns; however, visual inspection was necessary to confirm the results due to small band shifts. Besides, it is unknown which VNTR loci produced a certain DNA band. CGE/MLVF is superior to traditional MLVF because primers of each locus were labeled with different fluorescence. Only one limitation is that amplicons sdrC/sdrD/sdrE could not be differentiated due to the use of the same primers. CGE/MLVF also has the potential to produce portable results. In addition, clustering software used by CGE/MLVF (NTsyspc) is available and easy to use. However, bionumerics software is expensive and difficult to use.

In the present study, we showed that CGE/MLVF was the highest resolution typing method compared with MLST/SCC*mec*, *spa* typing and PFGE against epidemic MRSA. CGE/MLVF showed a strong discriminatory power mainly due to using the same set of primers as traditional MLVF. Previous studies have confirmed that the discriminatory power of traditional MLVF was found to be higher than those of MLST other MLVA methods, *spa* typing and other PCR-based methods [11,14–16]. Rivero-Pérez *et al.* [21] and Luczak-Kadluboska *et al.* [22] also reported that traditional MLVF was at least as discriminatory as PFGE when isolates had not been preselected.

Since the concordance levels between typing methods may vary corresponding to the collection of isolates, different studies of the correlation of traditional MLVF and other techniques have led to various conclusions [14,21,23]. Our study showed that CGE/MLVF, as well as traditional MLVF, was well concordant in cluster level comparing with PFGE and *spa* typing when using the similar coefficient of 0.905. Isolates clustering in the same CGE/MLVF CC were separated into the same PFGE cluster, *spa* type and STs. Isolates separated in different PFGE cluster, *spa* type and STs never clustered into the same CGE/MLVF cluster. However, the concordance between CGE/MLVF and PFGE and other typing methods at the subtyping level was low. Other studies comparing VNTR methods with PFGE also have shown much greater concordance between methods at the cluster level compared to subtyping level [11,14,24].

CGE/MLVF is more feasible to perform and needs no special equipment or skillful staff. In addition, high throughput and low time consumption (one workday for the entire typing procedure) make CGE/MLVF superior to PFGE (3 workdays) and many other methods for epidemiological monitoring in local hospitals. Moreover, the typing result is easy to explain, because any two DNA bands differing by more than 1 bp were considered different bands, and any two isolates differing by one or more bands were considered distinct types. Generally isolates differing by up to three bands by CGE/MLVF typing equals six bands difference by PFGE. It means that they belong to different clusters. In addition, CGE/MLVF needs no expensive reagent. The cost of CGE/MLVF is about 10 RMB/isolate (1.7 US dollar/isolate, including fluorescence labeled primer and electrophoresis cost), which is much lower than that of MLST (140 RMB/isolate, 23 US dollar/isolate) and PFGE (25 RMB/isolate, 4 US dollar/isolate). CGE/MLVF is suitable for typing the MRSA epidemic in China. ST239-III-t030 and ST239-III-t037 were the most prevalent nosocomial epidemic MRSA clones in China [25,26]. CGE/MLVF could easily distinguish between them due to different amplicon size of the *spa* locus, which is included in the CGE/MLVF typing. Moreover, CGE/MLVF can differentiate isolates of the same epidemic ST-SCC*mec*-*spa* clones in China. For example, CGE/MLVF differentiated 61 clinical isolates of the ST239-III-t030 clone (the most predominant clone in China) into eight CGE/MLVF types. Certain types only present in certain cities or hospitals, e.g., all CGE/MLVF type a2 isolates, only spread in the Jilin People’s Hospital. This demonstrates that CGE/MLVF could distinguish well the predominant MRSA clones in different regions.

CGE/MLVF has its limitations. One is that due to its being based on capillary gel electrophoresis, amplicons sizing over the detectable range will not be detected and that may decrease its resolution. Fortunately, very few isolates produce amplicons with sizing over the detectable range. The other is that comparison of CGE/MLVF genotype profiles is not as easy as MLVA (with a genotype profile form that is a code that can be stored in a database). However, high resolution, perfect reproducibility and ease of use make CGE/MLVF a good tool for genotyping.

## 4. Experimental Section

### 4.1. Bacterial Isolates

Strains MU3, MU50, USA300, COL, MW2 and thirty-seven previously well-characterized MRSA strains of diverse sequence types (STs) that were kindly provided by Dr. Matthew O’Sullivan and Professor Lyn G. Gilbert (Westmead Hospital, University of Sydney, Sydney, Australia) were used in this study (Table 1). A total of 116 non-replicate MRSA clinical isolates obtained from six hospitals distributed in three province of Northeast China between 2009 and 2010 were analyzed in the current study.

### 4.2. Extraction of Total DNA for PCR

Total DNA was prepared from 10 to 15 colonies lifted from blood agar plates incubated for 24 h at 37 °C and suspended in 300 μL of lysostaphy (40 U/L, BBI) for 30 min at 37 °C. The cell suspension was boiled for 15 min at 100 °C and then centrifuged for 5 min at a speed of 13,000 rpm. The suspension was transferred to a new tube. The DNA was quantified using a NanoDrop spectrophotometer (NanoDrop Technologies, Wilmington, DE, USA) at 260 nm.

### 4.3. Traditional MLVF

Traditional MLVF was performed as previously described [21]. The clustering dendrogram was generated by the unweighted-pair group method with arithmetic mean (UPGMA) using a Dice coefficient of similarity of 1.0%.

### 4.4. CGE/MLVF

#### 4.4.1. Multiplex PCRs

CGE/MLVF was a combination of VNTR multiplex PCRs of traditional MLVF and CGE. VNTR multiplex PCR using a set of PCR primers that Sabat *et al.* previously designed for traditional MLVF [13], whereas five VNTR loci were amplified in 2 multiplex PCRs. The two multiplex PCRs with different fluorescently labeled primer sets were prepared (see Table 1). Both panels contained a 2× EasyTaq PCR SuperMix (TransGen Biotech, Beijing, China) and 2 μL of DNA template in a final volume of 25 μL. The primer concentrations in panel 1 were 1.0 μM ClfA_F-HEX, ClfA_R, ClfB_F-FAM, ClfB_R, and 0.5 μM SdrCDE_F-ROX, SdrCDE_R and in panel 2 were 0.2 μM Spa_F-HEX, Spa_R, SspA_F-FAM, and SspA_R. Amplification of panel 1 and panel 2 were carried out as previously described by Sabat *et al.* [13]. After the PCR, 0.3 μL of reactions of panel 1 was mixed with 0.1 μL LIZ 1200 marker (Applied Biosystems, Foster City, CA, USA) and 10 μL of HiDi formamide (Applied Biosystems, Foster City, CA, USA), 0.3 μL of reactions of panel 2 was mixed with 0.1 μL LIZ 500 marker (Applied Biosystems, Foster City, CA, USA) and 10 μL of HiDi formamide. After heat denaturation at 95 °C for 2 min, the fragments produced in panel 1 and panel 2 were separated on an ABI 3730xl DNA sequencer (Applied Biosystems, Foster City, CA, USA) using the pop7_gs1200 and pop7_gs500 run module, respectively. The resulting files were imported and analyzed by using GeneMarker v 1.51 software (SoftGenetics, LLC, State College, PA, USA).

#### 4.4.2. Single PCR Verifying *spa* Locus

The PCR mix contained a 2× EasyTaq PCR SuperMix, 2 μL of DNA template, 0.2 μM Spa_F-HEX, Spa_R, in a final volume of 25 μL (TSINGKE, Beijing, China). Amplification was carried out in the same manner as multiplex PCR. After the PCR, 0.3 μL of reactions was mixed with 0.1 μL LIZ 500 marker and 10 μL of HiDi formamide. After heat denaturation at 95 °C for 2 min, the fragments were separated on an ABI 3730xl DNA sequencer using pop7_gs500 run module. The resulting files were imported and analyzed by using GeneMarker v 1.51 software (SoftGenetics, LLC, State College, PA, USA).

### 4.5. Spa Typing

Amplification of the variable X region of the *spa* gene was performed as described by Airesde-Sousa, *et al.* [27]. The *spa* types were assigned through the Ridom SpaServer (http://www.spaserver.ridom.de).

### 4.6. MLST and SCCmec

MLST was performed as described previously [28]. PCR fragments were purified and sequenced with an ABI 3700 sequencer. The sequences of the PCR products were compared with the existing sequences available on the MLST website (http://saureus.mlst.net) for *S. aureus*, and the allelic number was determined for each sequence. SCC*mec* was performed as described previously [29].

### 4.7. PFGE

PFGE typing of SmaI (Takara, Dalian, China)-digested DNA was performed through the use of a modification of a method previously described [30]. Briefly, MRSA colonies from overnight cultures were incorporated into agarose plugs. After bacterial lysis, genomic DNA was digested by using SmaI. PFGE was performed by clamped homogeneous electric field (CHEF) electrophoresis with a CHEF-mapper system (Bio-Rad Laboratories, Hercules, CA, USA). The fragments were separated with a linear ramped pulse time of 5 to 40 s over a period of 19 h at 14 °C. Gels were analyzed by using BioNumerics software v 6.10 (Applied Maths, Austin, TX, USA) using the Dice correlation coefficient. A dendrogram was generated by using the unweighted pair group method with arithmetic averages (UPGMA) with a tolerance of 1.5%.

### 4.8. Data Analysis

The Ridom EpiCompare software version 1.0 (http://www3.ridom.de/epicompare/) was used to calculate discriminatory power and concordance of the typing methods. The discriminatory power was estimated by Simpson’s index of diversity expressing the probability that two unrelated and different isolates sampled from the test population will be grouped as different subtypes by a specific typing method [31]. The 95% confidence intervals (CI) were calculated according to the method previously described by Grundmann *et al.* [32]. Non-overlapping confidence intervals were regarded as statistically significant differences in discriminatory power. The concordance between typing methods was assessed by adjusted Rand’s (*AR*) and Wallace’s (*W*) coefficients [33]. The AR coefficient indicates the global agreement between two methods, whereas the *W* coefficient shows the probability that two isolates classified as the same type by one method are also classified as the same type by another method.

For CGE/MLVF any two bands differing by more than one base pair were considered different bands, and any two isolates differing by one or more bands were considered distinct types. VNTR loci can be distinguished by different fluorescence. For clustering, the presence and absence of all alleles of every locus against each isolate were transformed to binary matrix and saved as Excel files and imported into NTsyspc software v 2.10 (Exeter Software, Setauket, NY, USA) and clustering dendrograms were produced by using UPGMA.

## 5. Conclusions

CGE/MLVF was cheaper, faster, easier to use in clinical practice than PFGE and many other tying methods, and was found to have identical discriminatory power as PFGE. The concordance between CGE/MLVF, PFGE and *spa* typing in group level was excellent. Consequently, CGE/MLVF could be a useful tool for control and prevention of MRSA in routine clinical microbiology laboratories.

## Supplementary Information



## Figures and Tables

**Table 1.** Primers used for CGE/MLVF of MRSA.

Panel	VNTR	Primer 1	Oligonucleotide sequences (5′ to 3′) 2	Size range (bp)	Size (bp) of Mu50

					In NCBI database
panel 1	*ClfA*	ClfA-F	GATTCTGACCCAGGTTCAGA-H	700–1500	1021
		ClfA-R	CTGTATCTGGTAATGGTTCTTT		

	*ClfB*	ClfB-F	ATGGTGATTCAGCAGTAAATCC-F	600–1000	832
		ClfB-R	CATTATTTGGTGGTGTAACTCTT		

	*Sdr*	SdrCDE-F	GTAACAATTACGGATCATGATG-R	400–1000	670/748/580
		SdrCDE-R	TACCTGTTTCTGGTAATGCTTT		

panel 2	*Spa*	Spa-F1	AGCACCAAAAGAGGAAGACAA-H	100–400	284
		Spa-R1	GTTTAACGACATGTACTCCGT		

	*SspA*	SspA-F	ATCMATTTYGCMAAYGATGACCA-F	100–200	173
		SspA-R	TTGTCTGAATTATTGTTATCGCC		

1 F, forward; R, reverse.

2 H, HEX; F, FAM; R, ROX.

**Table 2.** CGE/MLVF profiles and other typing results of the 42 well-characterized strains.

Strain	MLST	SCC-*mec*	*spa* type	CGE/MLVF	CGE/MLVF profile(bp)				
		
	ST			pattern	SspA	*spa*	sdrC&sdrD&sdrE	ClfB	ClfA
PC8	1	IV	t127	1	180.1	208.0	575.4/677.5 */683.6 *	903.5	1053.4
FH	1	IV	t127	2	123.8	207.8	575.4/784.7	802.9	1053.4
SJOG30	1	IV	t127	1	180.0	208.0	575.6/677.5 */683.6 *	903.9	1053.3
RPH85	1	IV	t127	3	180.0	207.5	575.4/677.5 */683.6 *	903.7	765.3
B827549	1	new	t1784	4	180.0	134.7	575.6/677.5	696.0	1053.6
SN39	1	new	t127	5	180.1	207.9	450.5/575.4/677.3	695.8	1053.2
RHH58	1	IV	t127	6	162.0	207.9	659.9	886.1	1071.4
RHH10	1	IV	t127	6	162.0	207.9	659.2	885.9	1071.4
MW2	1	IV	t128	7	123.7	232.4	575.4/629.6/671.5	909.9	1053.6
E804531	5	IV	t002	8	170.7	279.5	611.1/725.0/760.8	820.8	1160.8
BK2464	5	II	t002	9	170.5	278.6	599.9/611.1/683.0	731.1	926.3
MU3	5	II	t002	10	170.6	279.0	575.3/665.3/742.7	820.4	1016.5
MU50	5	II	t002	10	170.6	279.1	575.4/665.4/742.8	820.4	1016.8
B8-10	8	IV	t711	11	123.8	233.1	599.3/647.6/731.1	833.3	999.3
RPH2	8	NT	t190	12	152.4	208.1	599.4/611.4/725.1	647.4	957.0
IMVS67	8	V	t008	13	152.6	278.8	599.9/647.5/802.5	851.1	999.1
DEN-2988	8	II	t008	14	152.8	279.0	492.0	933.9	1011.0
USA300	8	IV	t008	15	152.4	279.0	599.3/647.4/731.0	851.2	1011.2
RPH81	239	III	t037	16	152.5	208.5	563.3 */575.9 */730.8	991.0 *	993.5 *
14176-5710	239	III	t1959	17	152.5	232.8	558.0/635.2/695.3	973.2	1011.4
K704540	239	III	t037	18	152.5	208.2	593.8/635.2/713.1	973.2	1011.5
ANS46	239	III	t037	18	152.4	208.0	593.7/635.1/713.0	972.8	1011.0
K711532	239	III	t037	19	152.5	208.4	593.8/617.2/713.1	972.9	1011.2
AH13	239	III	t037	20	152.5	208.4	569.7/724.7	991.0 *	993.5 *
RPAH18	239	III	t037	21	152.5	208.4	551.3/593.8/641.6	955.0 *	957.3 *
RPAH15	239	III	t037	22	152.4	208.3	551.5/594.0/713.3 *	703.2 *	1011.5 *
HDG2	239	III	t421	23	152.5	184.5	563.4/730.9/814.7	990.9 *	1011.0 *
HU25	239	III	t138	24	152.7	184.5	575.9/635.2/730.9	991.3 *	1011.4 *
AH1	128	III	t037	16	152.5	208.2	563.4 */576.1 */731.0	991.4 *	993.8 *
COL	250	I	t008	25	152.5	279.0	635.2 */647.8 */731.2	928.5	1011.4
CH16	22	IV	t032	26	207.1	419.5	611.3/712.7	881.9	1022.2
CH69	22	IV	t1963	27	188.8	326.4	611.3/712.7	881.9	1022.2
PAH58	30	IV	t019	28	216.1	233.0	617.1/693.7/718.8	990.8	1190.1
PAH1	30	IV	t019	29	216.1	233.0	521.8/599.1/693.8	990.8	1190.1
E822485	36	II	t018	30	216.0	301.9	563.3/603.9/683.1	810.3	1254.2
RPH74	45	V	t065	31	142.5	256.3	611.4/904.3 */915.9 *	1160.2	1111.4
J710566	new	V	t065	32	142.5	256.3	611.5/695.2/719.1	1192.8	1129.5
IP01M0181	59	IV	t216	33	188.0	232.7	527.9/599.1/724.9	1037.0	1248.7
C801535	78	NT	t325	34	226.3	278.3	468.4/700.0/802.8	862.1	1070.7
IP01M-2046	88	IV	t1958	35	152.5	256.1	468.6/581.2/784.7	808.4	1143.2
RBH98	93	IV	t202	36	170.4	233.5	593.0/720.0/1012.3	965.5	1137.8
137924492	93	IV	t202	36	170.4	233.3	592.9/720.0/1012.1	965.4	1137.2

Total types/patterns	15	7	20	36					

* amplicons of similar size against one strain could be differentiated by CGE/MLVF.

**Table 3.** Discrimination indices of compared methods against 116 clinical isolates.

Method	No. of clusters	No. of types/patterns	DIs	(95% CI)
PFGE	10	28	0.854	(0.818–0.89)
MLVF	11	28	0.855	(0.807–0.902)
CGE/MLVF	11	28	0.855	(0.807–0.902)
*spa* typing		11	0.623	(0.559–0.687)
MLST/SCC*mec*		8	0.517	(0.449–0.585)

PFGE types were grouped by UPGMA using similar cut-off value of 84%. MLVF types were grouped by UPGMA using similar cut-off value of 65%. CGE/MLVF types were grouped by UPGMA with similar coefficient 0.905.

**Table 4.** Correlations between four typing methods according to adjusted Rand’s coefficient and Wallace’s coefficient.

Typing method	Adjusted Rand’s coefficient				Wallace’s coefficient			
	
	*Spa* type	MLST/	PFGE	CGE/MLVF	*Spa* type	MLST/	PFGE	CGE/MLVF
	
		SCC*mec*	pattern/CC (CI 84% 1)	pattern/CC (SC 0.905 2)		SCC*mec*	pattern/CC (CI 84% 1)	pattern/CC (SC 0.905 2)
*Spa* type		0.786	0.441/0.977	0.438/0.988		1.000	0.388/0.986	0.385/0.986

MLST/SCC*mec*			0.31/0.786	0.308/0.776	0.78		0.303/0.781	0.301/0.77

PFGE pattern				0.384/0.449	1.000	1.000		0.472/1.000
PFGE CC (CI 84% 1)				0.438/0.989	0.986	1.000		0.385/0.986

CGE/MLVF pattern					1.000	1.000	0.476/1.000	
CGE/MLVF SC 0.905 2					1.000	1.000	0.393/1.000	

1 PFGE CC defined by the 84% cut-off value;

2 CGE/MLVF CC defined by the similar coefficient 0.905.
