# Supplementary material for: An Improved MLVF Method and Its Comparison with Traditional MLVF, spa Typing, MLST/SCCmec and PFGE for the Typing of Methicillin-Resistant Staphylococcus aureus
Source: Int J Mol Sci. 2014 Jan 8;15(1):725–42. doi: 10.3390/ijms15010725 (PMC3907834; doi:10.3390/ijms15010725)
Supplement: Supplementary file 1 [file ijms-15-00725-s001.pdf]

Supplementary Information

Table S1. *spa* typing results of 116 clinical isolates.

**strain**	***spa* repeat**	***spa* type**
461	R15-R12-R16-R02-R24-R24	t030
464	R15-R12-R16-R02-R24-R24	t030
468	R15-R12-R16-R02-R24-R24	t030
469	R15-R12-R16-R02-R24-R24	t030
472	R15-R12-R16-R02-R24-R24	t030
475	R15-R12-R16-R02-R24-R24	t030
478	R15-R12-R16-R02-R24-R24	t030
480	R15-R12-R16-R02-R24-R24	t030
485	R15-R12-R16-R02-R24-R24	t030
489	R15-R12-R16-R02-R24-R24	t030
490	R15-R12-R16-R02-R24-R24	t030
491	R15-R12-R16-R02-R24-R24	t030
494	R15-R12-R16-R02-R24-R24	t030
495	R15-R12-R16-R02-R24-R24	t030
498	R15-R12-R16-R02-R24-R24	t030
502	R15-R12-R16-R02-R24-R24	t030
503	R15-R12-R16-R02-R24-R24	t030
504	R15-R12-R16-R02-R24-R24	t030
508	R15-R12-R16-R02-R24-R24	t030
509	R15-R12-R16-R02-R24-R24	t030
513	R15-R12-R16-R02-R24-R24	t030
514	R15-R12-R16-R02-R24-R24	t030
517	R15-R12-R16-R02-R24-R24	t030
518	R15-R12-R16-R02-R24-R24	t030
521	R15-R12-R16-R02-R24-R24	t030
522	R15-R12-R16-R02-R24-R24	t030
523	R15-R12-R16-R02-R24-R24	t030
524	R15-R12-R16-R02-R24-R24	t030
525	R15-R12-R16-R02-R24-R24	t030
528	R15-R12-R16-R02-R24-R24	t030
529	R15-R12-R16-R02-R24-R24	t030
531	R15-R12-R16-R02-R24-R24	t030
532	R15-R12-R16-R02-R24-R24	t030
534	R15-R12-R16-R02-R24-R24	t030
535	R15-R12-R16-R02-R24-R24	t030
536	R15-R12-R16-R02-R24-R24	t030
537	R15-R12-R16-R02-R24-R24	t030
538	R15-R12-R16-R02-R24-R24	t030
541	R15-R12-R16-R02-R24-R24	t030
542	R15-R12-R16-R02-R24-R24	t030
544	R15-R12-R16-R02-R24-R24	t030
546	R15-R12-R16-R02-R24-R24	t030
548	R15-R12-R16-R02-R24-R24	t030
549	R15-R12-R16-R02-R24-R24	t030

Table S1. *Cont.*

strain	*spa* repeat	*spa* type
551	R15-R12-R16-R02-R24-R24	t030
553	R15-R12-R16-R02-R24-R24	t030
527	R15-R12-R16-R02-R24-R24	t030
539	R15-R12-R16-R02-R24-R24	t030
947	R15-R12-R16-R02-R24-R24	t030
950	R15-R12-R16-R02-R24-R24	t030
953	R15-R12-R16-R02-R24-R24	t030
959	R15-R12-R16-R02-R24-R24	t030
963	R15-R12-R16-R02-R24-R24	t030
966	R15-R12-R16-R02-R24-R24	t030
975	R15-R12-R16-R02-R24-R24	t030
981	R15-R12-R16-R02-R24-R24	t030
989	R15-R12-R16-R02-R24-R24	t030
1361	R15-R12-R16-R02-R24-R24	t030
1360	R15-R12-R16-R02-R24-R24	t030
1900	R15-R12-R16-R02-R24-R24	t030
1446	R15-R12-R16-R02-R24-R24	t030
473	R15-R12-R16-R02-R25-R17-R24	t037
474	R15-R12-R16-R02-R25-R17-R24	t037
476	R15-R12-R16-R02-R25-R17-R24	t037
477	R15-R12-R16-R02-R25-R17-R24	t037
486	R15-R12-R16-R02-R25-R17-R24	t037
492	R15-R12-R16-R02-R25-R17-R24	t037
493	R15-R12-R16-R02-R25-R17-R24	t037
555	R15-R12-R16-R02-R25-R17-R24	t037
1922	R15-R12-R16-R02-R25-R17-R24	t037
1926	R15-R12-R16-R02-R25-R17-R24	t037
1455	R15-R12-R16-R02-R25-R17-R24	t037
462	R26-R23-R17-R34-R17-R20-R17-R12-R17-R16	t002
463	R26-R23-R17-R34-R17-R20-R17-R12-R17-R16	t002
467	R26-R23-R17-R34-R17-R20-R17-R12-R17-R16	t002
481	R26-R23-R17-R34-R17-R20-R17-R12-R17-R16	t002
482	R26-R23-R17-R34-R17-R20-R17-R12-R17-R16	t002
484	R26-R23-R17-R34-R17-R20-R17-R12-R17-R16	t002
487	R26-R23-R17-R34-R17-R20-R17-R12-R17-R16	t002
499	R26-R23-R17-R34-R17-R20-R17-R12-R17-R16	t002
500	R26-R23-R17-R34-R17-R20-R17-R12-R17-R16	t002
501	R26-R23-R17-R34-R17-R20-R17-R12-R17-R16	t002
506	R26-R23-R17-R34-R17-R20-R17-R12-R17-R16	t002
507	R26-R23-R17-R34-R17-R20-R17-R12-R17-R16	t002
545	R26-R23-R17-R34-R17-R20-R17-R12-R17-R16	t002
552	R26-R23-R17-R34-R17-R20-R17-R12-R17-R16	t002
556	R26-R23-R17-R34-R17-R20-R17-R12-R17-R16	t002
558	R26-R23-R17-R34-R17-R20-R17-R12-R17-R16	t002
550	R26-R23-R17-R34-R17-R20-R17-R12-R17-R16	t002

Table S1. *Cont.*

strain	*spa* repeat	*spa* type
543	R26-R23-R17-R34-R34-R17-R20-R17-R12-R17-R16	t601
946	R26-R23-R17-R34-R17-R20-R17-R12-R17-R16	t002
954	R26-R23-R17-R34-R17-R20-R17-R12-R17-R16	t002
955	R26-R23-R17-R34-R17-R20-R17-R12-R17-R16	t002
956	R26-R23-R17-R34-R17-R20-R17-R12-R17-R16	t002
969	R26-R23-R17-R34-R17-R20-R17-R12-R17-R16	t002
973	R26-R23-R17-R34-R17-R20-R17-R12-R17-R16	t002
974	R26-R23-R17-R34-R17-R20-R17-R12-R17-R16	t002
980	R26-R23-R17-R34-R17-R20-R17-R12-R17-R16	t002
990	R26-R23-R17-R34-R17-R20-R17-R12-R17-R16	t002
1897	R26-R23-R17-R34-R17-R20-R17-R12-R17-R16	t002
1906	R26-R23-R17-R34-R17-R20-R17-R12-R17-R16	t002
1912	R26-R23-R17-R34-R17-R20-R17-R12-R17-R16	t002
1931	R26-R23-R17-R34-R17-R20-R17-R12-R17-R16	t002
1942	R26-R23-R17-R34-R17-R20-R17-R12-R17-R16	t002
1454	R26-R23-R17-R34-R17-R20-R17-R12-R17-R16	t002
1461	R26-R23-R17-R34-R17-R20-R17-R12-R17-R16	t002
1463	R26-R23-R17-R34-R17-R20-R17-R12-R17-R16	t002
1490	R26-R23-R17-R34-R17-R20-R17-R12-R17-R16	t002
511	R07-R23-R21-R17-R12-R23-R02-R12-R23	t796
512	R07-R16-R23-R02-R34	t899
520	R07-R12-R21-R17-R13-R34-R34-R34-R34-R33-R13	t2310
1449	R07-R23-R12-R12-R17-R20-R17-R12-R12-R17	t324
1480	R07-R23-R12-R12-R17-R20-R17-R12-R17	t664
1460	R26-R23-R17-R34-R17-R20-R17-R12-R16	t548
1466	R04-R20-R17-R20-R17-R25-R34	t437
965	R26-R23-R17-R34-R17-R20-R17-R12-R17-R16	t002

© 2014 by the authors; licensee MDPI, Basel, Switzerland. This article is an open access article distributed under the terms and conditions of the Creative Commons Attribution license (http://creativecommons.org/licenses/by/3.0/).
